# An Alternative to the Breeder’s and Lande’s Equations

**DOI:** 10.1534/g3.113.008433

**Published:** 2013-11-08

**Authors:** Bahram Houchmandzadeh

**Affiliations:** CNRS/University of Grenoble 1, LIPhy UMR 5588, Grenoble, F-38041, France

**Keywords:** linear response to selection, additive genetic effects, quantitative genetics

## Abstract

The breeder’s equation is a cornerstone of quantitative genetics, widely used in evolutionary modeling. Noting the mean phenotype in parental, selected parents, and the progeny by *E*(*Z*_0_), *E*(*Z_W_*), and *E*(*Z*_1_), this equation relates response to selection *R* = *E*(*Z*_1_) − *E*(*Z*_0_) to the selection differential *S* = *E*(*Z_W_*) − *E*(*Z*_0_) through a simple proportionality relation *R* = *h*^2^*S*, where the heritability coefficient *h*^2^ is a simple function of genotype and environment factors variance. The validity of this relation relies strongly on the *normal* (Gaussian) distribution of the parent genotype, which is an unobservable quantity and cannot be ascertained. In contrast, we show here that if the fitness (or selection) function is Gaussian with mean *μ*, an alternative, exact linear equation of the form *R*′ = *j*^2^*S*′ can be derived, regardless of the *parental genotype* distribution. Here *R*′ = *E*(*Z*_1_) − *μ* and *S*′ = *E*(*Z_W_*) − *μ* stand for the mean phenotypic *lag* with respect to the mean of the fitness function in the offspring and selected populations. The proportionality coefficient *j*^2^ is a simple function of selection function and environment factors variance, but *does not* contain the genotype variance. To demonstrate this, we derive the exact functional relation between the mean phenotype in the selected and the offspring population and deduce all cases that lead to a linear relation between them. These results generalize naturally to the concept of *G* matrix and the multivariate Lande’s equation $\Delta \bar{z}=GP^{-1}S$. The linearity coefficient of the alternative equation are not changed by Gaussian selection.

The breeder’s equation for the evolution of quantitative traits for additive genetic effects, introduced by Lush (Lush 1943), is widely used both in artificial and natural selection theory and experiments (Lande 1976; Falconer and Mackay 1995; Lynch and Walsh 1998; Heywood 2005) and appears in all textbooks of quantitative genetic. This equation can be stated as follow: consider a continuous phenotypic trait *Z* subject to selection. Noting the mean phenotype in parental, selected parents and the progeny by *E*(*Z*_0_), *E*(*Z_W_*) and *E*(*Z*_1_), we can define the selection differential *S* = *E*(*Z_w_*) − *E*(*Z*_0_) and the response *R* = *E*(*Z*_1_) − *E*(*Z*_0_). The scalar breeder’s equation reads *R* = *h*^2^*S* and ascertains that the response to selection and the selection differential are related through a proportionality relation that is the ratio of genotype to phenotype variances, *h*^2^. The equation naturally extends to selection on multiple traits and its vectorial version reads $\Delta \bar{z}=GP^{-1}S$

Use of the breeder’s equation and its underlying assumptions has been criticized by many authors (Kruuk 2004; Heywood 2005; Pigliucci 2006; Gienapp *et al.* 2008; Pemberton 2010). One fundamental assumption of the breeder’s equation is the normal (Gaussian) distribution of the breeding value (genotype) *and* environment factors. Authors who demonstrate the linear relation (Kimura and Crow 1978; Lande 1979; Lande and Arnold 1983; Nagylaki 1992; Falconer and Mackay 1995; Lynch and Walsh 1998; Crow and Kimura 2009) assume normal distribution for the aforementioned quantities or the analogous hypothesis of linearity of the parent−offspring regression (see Appendix/Parent−offspring regression). When this assumption is relaxed, the breeder’s equation is no longer valid, and one has to resort to a system of hierarchical moment (or alternatively, cumulant) equations to describe the changes; in general, this system is not closed, and the moments of a given order depend on moments of higher order (Turelli and Barton 1990).

The assumption of a Gaussian distribution of the genotype can be criticized on several grounds (Pigliucci and Schlichting 1997; Pigliucci 2006; Geyer and Shaw 2008). For example, the very act of selection causes the genotype distribution to deviate from a Gaussian (Turelli and Barton 1990; Turelli and Barton 1994) (see also equation 6 below). Another important case is when the genotype is a cross between different breeds due to external gene flow or the breeder’s scheme. In many cases, the phenotype can have a bell shape and thus is assumed to be Gaussian, when the genotype is indeed far from it (see, for example, Figure 2A). It is sometimes argued that even if the breeding value does not follow a normal distribution, a scale can be used to restore it to a normal distribution. Such a scale, however, will also distort the distribution of environment factors and the assumptions of breeder’s equation are violated even in this case.

For *additive* genetic effects and in the absence of epistasis and dominance, I derive here a precise functional relation between the mean of the trait in the selected subpopulation and in their progeny for the general case. The mathematical formulation is close to the framework used by many authors such as Slatkin, Lande and Karlin (Slatkin 1970; Karlin 1979; Lande 1979). I then use a standard tool of functional analysis, the Fourier transform (FT), to deduce all the cases that lead to a linear relation between the response *R* and the selection differential *S*, regardless of the selection function. These cases imply a precise form of the distributions of genotype and environment factors, and I show that the proportionality factor between *R* and *S* is the heritability coefficient *h*^2^ only if these distributions are *normal*.

The genotype, however, is not observable or controllable, and its normal distribution cannot be assumed *a priori*. I show that if instead of the genotype, the *fitness function* and environment factors are Gaussian, then a new proportionality relation can be obtained in the form of

$$R^{\prime }=j^{2}S^{\prime }$$ (1)

*regardless* of the genotype distribution. Noting the mean of the Gaussian selection function by *μ*, *R*′ = *E*(*Z*_1_) − *μ* and *S*′ = *E*(*Z_W_*) − *μ* are the mean phenotypic lag with respect to the mean of the fitness function of the progeny and the selected population (Figure 1). As *E*(*Z*_1_), *E*(*Z_W_*), and *μ* are all measurable, *R*′ and *S*′ are both measurable in the same way as *R* and *S* are. The *j*^2^ coefficient contains only the width of the fitness function and environment factors. The use of a Gaussian selection function, both in artificial and natural selection (as an approximation of stabilizing selection), is widespread (Lewontin 1964; Lande 1976; Kimura and Crow 1978; Zhang and Hill 2010) and the aforementioned relationship is potentially as useful as the standard breeder’s equation.

**Figure 1:**
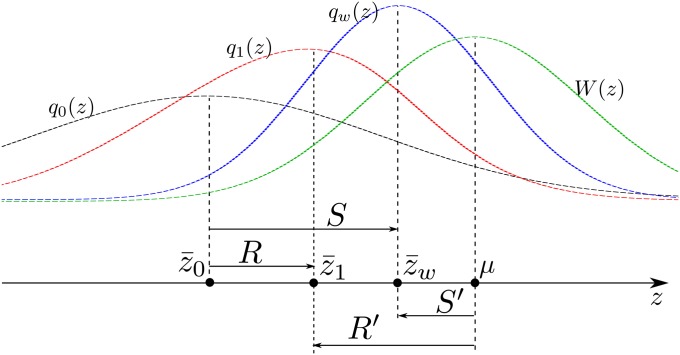
Schematic representation of the selection lag *S*′, the response lag *R*′, and their relation to the selection differential *S* and the response *R*. The mean phenotype of parental generation ${\bar{z}}_{0}$, selected population ${\bar{z}}_{w}$, the progeny ${\bar{z}}_{1}$, and the peak of selection function *μ* are represented on the phenotype axis *z*. Dashed curves represent a sketch of the distributions of parental phenotype *q*_0_(*z*), selected parents *q_w_*(*z*), the progeny *q*_1_(*z*), and the selection function *W*(*z*).

The advantage is more critical when the breeder’s or Lande’s equations are used in long-term evolution, where the variance of the genotype (or the *G* matrix) also varies and *h*^2^ cannot be assumed to remain constant (Gavrilets and Hastings 1995; Pigliucci and Schlichting 1997; Roff 2000) ; in contrast, the relation (1) remains valid if each round of selection uses a Gaussian fitness function.

The aforementioned results generalize naturally to multivariate trait selection where the alternative Lande’s equation is

$$R^{\prime }=\left(\Omega +E\right)\Omega^{-1}S^{\prime }$$ (2)

where **R**′ and **S**′ are the vectorial phenotype lag, and **Ω** and *E* are the covariance matrices of the fitness function and the environment respectively.

This article is organized as follows: in the *Results* section, I first derive the general functional relationship between *R* and *S*; the second subsection is devoted to all the cases where these two quantities can be linearly related, including the special case of the breeder’s equation. The alternative breeder’s equation is derived in the third subsection, and all the results are generalized to selection on multiple traits in the fourth subsection. The aforementioned results are put into perspective in the *Discussion* section. Technical details, such as the use of FTs, are treated in the Appendix.

## Results

### General results

Consider a continuous phenotype *Z*, which is the result of additive genetic effect *Y* and the environment *ξ* (Fisher 1918; Lynch and Walsh 1998; Visscher *et al.* 2008)

Z=Y+ξ

The term *environment* encompasses here any source of *noise* that causes the observed phenotype *z* to deviate from the (unobserved) breeding value *y* (Wright 1920; Lynch and Walsh 1998; Raj and van Oudenaarden 2008). In the following, the population distribution of the breeding value (genotype) and its variance in the parental generation are denoted *p*_0_(*y*) and $\sigma_{A}^{2}$. The environment effect is captured by the distribution law *f*(*z*|*y*), the probability density of observing phenotype *z* with the given genotype *y*. We will suppose that *f* is a symmetric function of its argument of the form *f*(*z*|*y*) = *f*(*z* − *y*) and denote its width by $\sigma_{E}^{2}$.

A subpopulation among the parental generation is selected according to a fitness or selection function *W*(*z*), the proportion of phenotypes in [*z*, *z* + *dz*] to be selected for the production of the next generation. The selected individuals produce offspring which will constitute the next generation. As we will show herein, the response *R* (the mean of the phenotype trait in the offspring) and the selection differential *S* (the mean of the phenotype trait in the selected parents) are given by

$$R=E\left(Z_{1}\right)=\frac{1}{\bar{W}}{\int \int }_{{\mathbb{R}}^{2}}yp_{0}\left(y\right)W\left(z\right)f\left(z-y\right)dydz$$ (3)

$$S=E\left(Z_{w}\right)=\frac{1}{\bar{W}}{\int \int }_{{\mathbb{R}}^{2}}zp_{0}\left(y\right)W\left(z\right)f\left(z-y\right)dydz$$ (4)

where $\bar{W}$ is the mean fitness of parental generation. Equations (3) and (4) are used, for example, by Lande (1979), although their derivation there depended on the normal distribution of the genotype. I derive these equations here for the more general case.

Before going into the details of calculations, note that the genotype distribution *p*_0_(*y*) and the selection function *W*(*z*) play a symmetric role in the aformentioned expressions. In the following sections, we will explore specific functional forms of *p*_0_(*y*) and *W*(*z*), which lead to a linear relationship between *R* and *S*. Because of the symmetric role of these two functions however, once a particular relation is obtained for a specific form of *p*_0_(*y*) regardless of *W*(*z*), an analogous relationship can be obtained for a similar form of *W*(*z*) regardless of *p*_0_(*y*). This is what leads us to an alternative form of the breeder’s equation.

Let us now derive the equations (3,4). We note that the distribution of the phenotype *Z* in the parental generation is given by

$$q_{0}\left(z\right)=\int_{\mathbb{R}}p_{0}\left(y\right)f\left(z|y\right)dy$$ (5)

We will denote its variance by $\sigma_{P}^{2}$.

The distribution of the phenotype *z* in the parental population selected according to the fitness function *W*(*z*) is

$$q_{w}\left(z\right)=\frac{1}{\bar{W}}q_{0}\left(z\right)W\left(z\right)$$

where $\bar{W}$ is the mean fitness of the parental generation

$$\begin{array}{c} \bar{W}=\int_{\mathbb{R}}q_{0}\left(z\right)W\left(z\right)dz\\ ={\int \int }_{{\mathbb{R}}^{2}}p_{0}\left(y\right)W\left(z\right)f\left(z|y\right)dydz \end{array}$$

The genotype distribution of the selected population is (Turelli and Barton 1994)

$$p_{w}\left(y\right)=\frac{1}{{\bar{W}}^{†}}\int_{\mathbb{R}}p_{0}\left(y\right)f\left(z|y\right)W\left(z\right)dz$$ (6)

$$=\frac{1}{{\bar{W}}^{†}}p_{0}\left(y\right)W^{†}\left(y\right)$$ (7)

where

$$W^{†}\left(y\right)=\int_{\mathbb{R}}W\left(z\right)f\left(z|y\right)dz$$ (8)

is the *genotype* fitness function, *i.e.*, the convolution of the phenotype fitness function by the environment factors. ${\bar{W}}^{†}$ is the mean genotype fitness:

$$\begin{array}{c} {\bar{W}}^{†}=\int_{\mathbb{R}}p_{0}\left(y\right)W^{†}\left(y\right)dy\\ ={\int \int }_{{\mathbb{R}}^{2}}p_{0}\left(y\right)W\left(z\right)f\left(z|y\right)dydz \end{array}$$

Note that $\bar{W}={\bar{W}}^{†}$ as both these quantities are defined by the same double integration over the domains of *y* and *z*.

For a large, randomly mating population, reproduction gives for the distribution of breeding values in the next generation (Slatkin 1970; Karlin 1979; Bulmer 1985; Turelli and Barton 1994)

$$p_{1}\left(y\right)={\int \int }_{{\mathbb{R}}^{2}}p_{w}\left(y_{a}\right)p_{w}\left(y_{b}\right)L\left(y-\left(y_{a}+y_{b}\right)/2\right)dy_{a}dy_{b}$$

The exact form of the probability density L(y) that captures the inheritance process (recombination, segregation, …) is not important here; Turelli and Barton (1994), for example, use a normal distribution for L(y) in the framework of the infinitesimal model. For our purpose, it is enough to suppose that the mean of the distribution L(y) is zero, *i.e.*, $\int_{y}yL\left(y\right)dy=0$ which is valid in the absence of dominance and epistasis effects (Turelli and Barton 1990) (see also *Appendix/Segregation density function*).

The phenotype distribution of the progeny is

$$q_{1}\left(z\right)=\int_{\mathbb{R}}p_{1}\left(y\right)f\left(z|y\right)dy$$ (9)

We now make the further assumption that (1) the environment and genotype are independent random variables, so that f(z|y)=f(z−y) and therefore the variances are additive: $\sigma_{P}^{2}=\sigma_{A}^{2}+\sigma_{E}^{2}$ and (2) environment effects are of zero mean $\left(\int_{x}xf\left(x\right)dx=0\right)$ and symmetric (*f*(−*x*) = *f*(*x*)). An environmental noise with such a distribution law does not change the mean of the random variable: *E*(*Z*) = *E*(*Y* + *ξ*) = *E*(*Y*). Therefore, the mean phenotype of the offspring is

$$\begin{array}{c} R=E\left(Z_{1}\right)=E\left(Y_{1}\right)\\ =\int_{\mathbb{R}}yp_{1}\left(y\right)dy\\ =\left(1/2\right)\iint_{{\mathbb{R}}^{2}}\left(y_{a}+y_{b}\right)p_{w}\left(y_{a}\right)p_{w}\left(y_{b}\right)dy_{a}dy_{b}\;\\ =\int_{\mathbb{R}}yp_{w}\left(y\right)dy \end{array}$$ (10)

$$=\frac{1}{\bar{W}}{\int \int }_{{\mathbb{R}}^{2}}yp_{0}\left(y\right)W\left(z\right)f\left(z-y\right)dydz$$ (11)

which is equation (3). Note that the first lines of the above equations merely state that the *expectations* of the breeding’s value of parent and offspring are equal for purely additive traits.

On the other hand, the mean phenotype of the selected parents is

$$\begin{array}{c} S=E\left(Z_{w}\right)=\int_{\mathbb{R}}zq_{w}\left(z\right)dz\\ =\frac{1}{\bar{W}}\int_{\mathbb{R}}zq_{0}\left(z\right)W\left(z\right)dz\\ =\frac{1}{\bar{W}}{\int \int }_{{\mathbb{R}}^{2}}zp_{0}\left(y\right)W\left(z\right)f\left(z-y\right)dydz \end{array}$$ (12)

which is equation (4).

For an asexually reproducing organism, or for a sexually reproducing population which remains at Hardy-Weinberg equilibrium after selection-reproduction, we would have *p*_1_(*y*) = *p_w_*(*y*) ; this would again lead to the same equation (10) and the same response (11). The conditions for the existence of multilocus Hardy-Weinberg equilibrium were analyzed by Karlin and Liberman (1979a,b), who concluded that for additive traits, the equilibrium is stable for a wide range of recombination distributions. The general relation between *R* and *S* can also be studied in the context of the Price equation. A detailed study of this relation has been performed by Heywood (2005).

### Conditions for proportionality of ***R*** and ***S***

The relations (3) and (4) show that the selection differential *S* and the response *R* to it are related through a functional equation involving three factors: genotype distribution, the selection function and the environmental noise. It is far from obvious that *R* and *S* could be proportional, a question we will investigate by using FTs.

FTs in functional analysis play a role analogous to logarithms in algebra. They are useful for clarifying the *R* − *S* relation, where we can transform the double integrations into simple ones. The FT of the function *u*(*x*) is the function $\tilde{u}\left(k\right)$ defined as (see *Appendix/Fourier Transforms*)

$$\tilde{u}\left(k\right)=\int_{-\infty }^{\infty }e^{-ikx}u\left(x\right)dx.$$

For example, the FT of the function *u*(*x*) = exp(−*α*|*x*|) is $\tilde{u}\left(k\right)=2\alpha /\left(\alpha^{2}+k^{2}\right)$. Part of the usefulness of FT is due to the fact that they transform convolution products into simple products: given two functions *u*(*x*) and *v*(*x*) and their convolution product *h*(*z*):

$$h\left(z\right)=\int_{-\infty }^{\infty }u\left(x\right)v\left(z-x\right)dx$$

the relation between their FT is a simple product:

$$\tilde{h}\left(k\right)=\tilde{u}\left(k\right)\tilde{v}\left(k\right)$$

As the general relations (11) and (12) involve convolutions, FT proves to be very useful in their handling. Using the various properties of FT (see *Appendix/Fourier Transforms*), the relation between *R* and *S* in the Fourier space reads:

$$R=\frac{i}{2\pi \bar{W}}\int_{\mathbb{R}}{\tilde{W}}^{*}\left(k\right)\frac{d}{dk}\left[{\tilde{p}}_{0}\left(k\right)\right]\tilde{f}\left(k\right)dk$$ (13)

and

$$\begin{array}{c} S=\frac{i}{2\pi \bar{W}}\int_{\mathbb{R}}{\tilde{W}}^{*}\left(k\right)\frac{d}{dk}\left[{\tilde{p}}_{0}\left(k\right)\tilde{f}\left(k\right)\right]dk\\ =R+\frac{i}{2\pi \bar{W}}\int_{\mathbb{R}}{\tilde{W}}^{*}\left(k\right){\tilde{p}}_{0}\left(k\right)\frac{d}{dk}\left[\tilde{f}\left(k\right)\right]dk \end{array}$$ (14)

where the mean fitness $\bar{W}$ is itself defined in Fourier space as

$$\bar{W}=\frac{1}{2\pi }\int_{\mathbb{R}}{\tilde{W}}^{*}\left(k\right){\tilde{p}}_{0}\left(k\right)\tilde{f}\left(k\right)dk.$$

Here a* designate the complex conjugate of a, i^2^ = −1 and we have set the origin of the breeding values at its mean in the parental population, *i.e.*, $\int_{\mathbb{R}}yp_{0}\left(y\right)dy=0$.

In general, the FT of a function is complex. However, as the function in direct space here are real, it can be shown that expressions (13) and (14) are indeed real; the fact that *i* appears in these expression insures this fact (see *Appendix/Fourier Transforms*). It is worthwhile to consider a particular case to clarify the above expressions. The detailed computations for a truncation selection in which breeding value and environmental factors are normally distributed are provided in *Appendix/Truncation* selection.

We see from equations (13) and (14) that *S* and *R* can be proportional if the second term of the r.h.s. of equation (14) is proportional to R; this will be true, regardless of the selection function W, if

$${\tilde{p}}_{0}\left(k\right)\frac{d\tilde{f}\left(k\right)}{dk}=a\frac{d{\tilde{p}}_{0}\left(k\right)}{dk}\tilde{f}\left(k\right)$$ (15)

where *a* is an arbitrary constant. Equation (15) is the necessary and sufficient condition that defines the functional shape of the genotype distribution and the environment noise compatible with the proportionality of *R* and *S* regardless of the selection function. If condition (15) is fulfilled, then

$$R={\left(1+a\right)}^{-1}S$$

On the other hand, equation (15) can be seen as a differential equation whose solution is given by

$$\tilde{f}\left(k\right)=b{\tilde{p}}_{0}{\left(k\right)}^{a}$$ (16)

where *b* is another arbitrary constant. Let us consider some particular case where the aforementioned relation is obeyed.

### Normal distributions

If $\tilde{f}\left(k\right)$ and ${\tilde{p}}_{0}\left(k\right)$ are both Gaussians, *i.e.*,

$$\tilde{f}\left(k\right)=\text{exp}\left(-\sigma_{E}^{2}k^{2}/2\right)$$

$${\tilde{p}}_{0}\left(k\right)=\text{exp}\left(-\sigma_{A}^{2}k^{2}/2\right)$$

then the relation (16) is satisfied by

$$a=\sigma_{E}^{2}/\sigma_{A}^{2}$$

and we retrieve the usual breeder’s equation *R* = *h*^2^*S* where $h^{2}=\sigma_{A}^{2}/\left(\sigma_{A}^{2}+\sigma_{E}^{2}\right)$. Of course, if $\tilde{f}\left(k\right)$ and ${\tilde{p}}_{0}\left(k\right)$ are of the above form, their inverse FTs represent normal distributions of width *σ_E_* and *σ_A_* respectively (see *Appendix/Fourier Transforms*).

### Stretched exponentials

We see, however, that even if the strict condition (16) is fulfilled, the proportionality constant need not be *h*^2^. Consider, for example, the class of stretched exponential functions *φ*(*k*) = exp(−|*k*|*^α^*), which generalizes Gaussians (case *α* = 2). Set $\tilde{f}\left(k\right)=\varphi \left(\sigma_{E}k\right)$, ${\tilde{p}}_{0}\left(k\right)=\varphi \left(\sigma_{A}k\right)$. The inverse FT of these functions gives the distribution of the genotype *Y* and environment effect *E* and it is straightforward to show that as for the Gaussian case, $\text{Var}\left(E\right)/\text{Var}\left(Y\right)=\sigma_{E}^{2}/\sigma_{A}^{2}$. Condition (16) however is satisfied this time with $a=\sigma_{E}^{\alpha }/\sigma_{A}^{\alpha }$ and therefore the realized heritability *h^α^* = *R*/*S* is

$$h^{\alpha }=\frac{\sigma_{A}^{\alpha }}{\sigma_{A}^{\alpha }+\sigma_{E}^{\alpha }}$$

**Figure 2:**
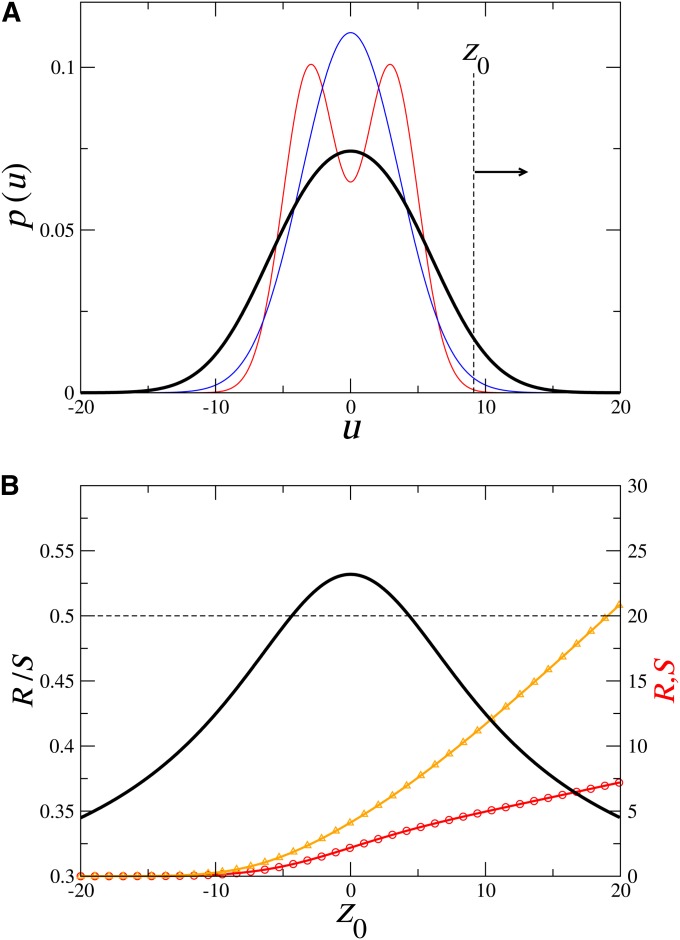
A simple example in which *R*/*S* ≠ *h*^2^. (A) The parental breeding value distribution (thin red line) is a double Gaussian *p*_0_(*y*) = (N(*m*, *s*; *y*) + N(−*m*, *s*; *y*))/2; the environmental effects distribution (thin blue line) follows a normal distribution *f*(*x*) = N(0, *σ_E_*; *x*). The phenotype distribution *q*(*z*) (equation 5), (thick black line), has the appearance of a normal distribution. The result of a truncation selection, selecting only and all individuals with phenotype value > *z*_0_ is shown in (B). (B) Right scale: The Response *R* (red line, circle) and the Selection differential *S* (orange line, triangle) as a function of the truncation selection *z*_0_. Left scale: the value of *R*/*S* (thick black line) as a function of *z*_0_ and its comparison to *h*^2^ (thin dashed line). All integrations (equations 11 and 12) can be performed exactly for this case: *R*(*z*_0_) and *S*(*z*_0_) are combination of Gaussian and erf(*z*_0_) functions, their exact expressions are given in *Appendix/Computation of truncation* selection. The parameters of the figures are *m* = 3, *s* = 2 and $\sigma_{E}=\sigma_{A}=\sqrt{m^{2}+s^{2}}=\sqrt{13}$, therefore *h*^2^ = 1/2.

The aforementioned examples were to emphasize the fact that selection-independent proportionality is achieved only for particular pairs of genotype/environment distributions. In general, as shown in Figure 2, the realized heritability is not constant and depends critically on the selection function *W*(*z*).

### Alternative breeder’s equation

Optimal phenotypic selection approximated by Gaussians has been considered by many authors both in artificial (as early as Lush 1943) and in natural selection (as early as Wright 1935; Haldane 1954) and it is widespread in the literature (Lewontin 1964; Lande 1976; Kimura and Crow 1978; Karlin and Liberman 1979a; Zhang and Hill 2010). If the selection function is Gaussian, a new linear relation can be extracted from the general relations (3) and (4), *regardless* of the (unobservable) breeding value distribution.

Note that a symmetric role is played by *W*(*z*) and *p*_0_(*y*) in the general expressions (3) and (4). Hence permuting their role will lead us, following the same line of arguments, to deduce all linear cases regardless of genotype. Equations (3) and (4) are obtained by multiplying the function *F*(*y*, *z*) = *W*(*z*)*p*_0_(*y*)*f*(*z* − *y*) either by *y* or *z* and integrating over ℝ^2^. To obtain the breeder’s equation of the previous section, we wrote the integration over the *y* variable as a convolution product and performed the FT on the *z* variable.

On the other hand, we could have proceeded by writing equations (3) and (4) first as a convolution product on *z* and then perform a FT on the variable *y* (see *Appendix/Fourier Transform*). In this case, we get

$$S=\frac{i}{2\pi \bar{W}}\int_{\mathbb{R}}{\tilde{p}}^{*}\left(k\right)\frac{d}{dk}\left[\tilde{W}\left(k\right)\right]\tilde{f}\left(k\right)dk$$ (17)

and

$$R=\frac{i}{2\pi \bar{W}}\int_{\mathbb{R}}{\tilde{p}}^{*}\left(k\right)\frac{d}{dk}\left[\tilde{W}\left(k\right)\tilde{f}\left(k\right)\right]dk$$ (18)

The arguments of the previous section can be repeated. Let us center the selection function by setting *W*(*z*) = *W_c_*(*z* − *μ*) where

$$\mu =\int_{\mathbb{R}}zW\left(z\right)dz$$

Then

$$S^{\prime }=\left(S-\mu \right)=\frac{i}{2\pi \bar{W}}\int_{\mathbb{R}}{\tilde{p}}^{*}\left(k\right)e^{-ik\mu }\frac{d}{dk}\left[\tilde{W_{c}}\left(k\right)\right]\tilde{f}\left(k\right)dk$$ (19)

and

$$R^{\prime }=\left(R-\mu \right)=\frac{i}{2\pi \bar{W}}\int_{\mathbb{R}}{\tilde{p}}^{*}\left(k\right)e^{-ik\mu }\frac{d}{dk}\left[\tilde{W_{c}}\left(k\right)\tilde{f}\left(k\right)\right]dk$$ (20)

The quantities *S*′ and *R*′ are alternative selection differential and response and represent the *lag* with respect to the mean of the selection function (Figure 1). In the case in which the selection function and the environment factors are both normally distributed with width *σ_W_* and *σ_E_*, a repetition of the arguments of the previous sections leads to

$$R^{\prime }=j^{2}S^{\prime }$$ (21)

where

$$j^{2}=\frac{\sigma_{W}^{2}+\sigma_{E}^{2}}{\sigma_{W}^{2}}$$

We stress that relation (21) is obtained *regardless* of the unknown genotype distribution *p*_0_(*y*).

The alternative breeder’s equation (21) may seem unusual as it does not contain the genetic variance. Such a result may seem at first glance in contradiction with our basic understanding of the selection process. Fisher fundamental’s theorem for example explicitly relates the rate of increase in fitness to the genetic variance. There is, however, no contradiction: Both *R*′ and *S*′ *are dependent* on the genetic variance, as can be seen in the general equations (3) and (4) ; however, their *ratio*, *i.e.*, the coefficient of the linear equation (21) relating them, is free of genetic variance. A similar situation occurs for the classical breeder’s equation, where both *R* and *S* depend on the selection function *W*(*z*) but their *ratio* contains only the heritability coefficient, independently of *W*(*z*).

Equation (21) has been obtained through the tools of functional analysis and its demonstration may seem a little abstract. It is worthwhile to further illustrate this equation by considering few examples where the computations can be carried out explicitly. Let us designate the normally distributed selection function *W*(*z*) and the environment factors as:

$$W\left(z\right)=N\left(\mu ,\sigma_{W};z\right)$$

$$f\left(x\right)=N\left(0,\sigma_{E};x\right)$$

where $N\left(a,b;u\right)=\left(1/\sqrt{2\pi }b\right)\text{exp}\left(-{\left(u-a\right)}^{2}/2b^{2}\right)$.

### No genetic variance

The first example we consider is the extreme case in which there is no genetic variance (σA = 0) in the parental generation. The distribution of the breeding value then becomes a Dirac’s delta function p0(y) = δ(y). The basic rule of Dirac’s delta, *i.e.*, $\int_{I}\delta \left(y\right)\varphi \left(y\right)dy=\varphi \left(0\right)$ reduces the double integrations of equations (11) and (12) to simple integrations which involve only Gaussian functions. Note that for the general case, reduction of double integration to simple one was achieved by the use of FTs. The value of R and S are therefore readily obtained in this case:

$$\bar{W}=\int_{\mathbb{R}}W\left(z\right)f\left(z\right)dz=\frac{1}{\sqrt{2\pi }}\frac{\text{exp}\left(-\frac{\mu^{2}}{2\left(\sigma_{E}^{2}+\sigma_{W}^{2}\right)}\right)}{\sqrt{\sigma_{E}^{2}+\sigma_{W}^{2}}}$$ (22)

R=0 (23)

$$S=\frac{1}{\bar{W}}\int_{\mathbb{R}}zW\left(z\right)f\left(z\right)dz=\mu \frac{\sigma_{E}^{2}}{\sigma_{W}^{2}+\sigma_{E}^{2}}$$ (24)

As expected, in the absence of genetic variance, there is no response to selection. The response and selection lag *R*′ and *S*′ read:

$$R^{\prime }=R-\mu =-\mu$$

$$S^{\prime }=S-\mu =-\frac{\sigma_{W}^{2}}{\sigma_{W}^{2}+\sigma_{E}^{2}}\mu$$

Therefore, *R*′ = *j*^2^*S*′, and equation (21) is verified. This example shows explicitly that there is no contradiction between the alternative breeder’s equation and Fisher’s fundamental theorem.

### Gaussian breeding values distribution

Let us now consider a less-extreme case in which there exists a normal genetic variability

$$p_{0}\left(y\right)=N\left(0,\sigma_{A};y\right)$$

The double integrations (11,12) giving *S* and *R* can again be carried out exactly, as all the integrands are Gaussian:

S=αμ (25)

$$R=\alpha h^{2}\mu$$ (26)

where *α* = (*j*^2^ − 1)/(*j*^2^ − *h*^2^). Therefore,

$$\frac{R^{\prime }}{S^{\prime }}=\frac{R-\mu }{S-\mu }=\frac{\alpha h^{2}-1}{\alpha -1}=j^{2}$$

and equation (21) is verified.

Note that in the aforementioned case, *R* and *S* are both *proportional* to the mean of the selection function *μ*. Applying a Gaussian selection function can therefore be used as a test of the normal distribution of the breeding values.

### Non-Gaussian breeding values distribution

Let us now consider a case in which parental breeding values are not normally distributed but are concentrated around two particular values:

$$p_{0}\left(y\right)=\frac{1}{2}\left(\delta \left(y-\sigma_{A}\right)+\delta \left(y+\sigma_{A}\right)\right)$$ (27)

and therefore *E*(*Y*_0_) = 0 and $\text{Var}\left(Y_{0}\right)=\sigma_{A}^{2}$. The computation of the expressions (11) and (12) can be again carried out exactly:

$$S=\frac{\mu \sigma_{E}^{2}+\sigma_{A}\sigma_{W}^{2}\;\tanh\left(\frac{\sigma_{A}\mu }{\sigma_{W}^{2}+\sigma_{E}^{2}}\right)}{\sigma_{W}^{2}+\sigma_{E}^{2}}$$

$$R=\sigma_{A}\;\text{tanh}\left(\frac{\sigma_{A}\mu }{\sigma_{W}^{2}+\sigma_{E}^{2}}\right)$$

We note that in this case, the ratio *R*/*S* ≠ *h*^2^ and the classical breeder’s equation does not hold. The alternative breeder’s equation however is again verified:

$$\frac{R^{\prime }}{S^{\prime }}=\frac{R-\mu }{S-\mu }=\frac{\sigma_{W}^{2}+\sigma_{E}^{2}}{\sigma_{W}^{2}}=j^{2}$$

**Figure 3:**
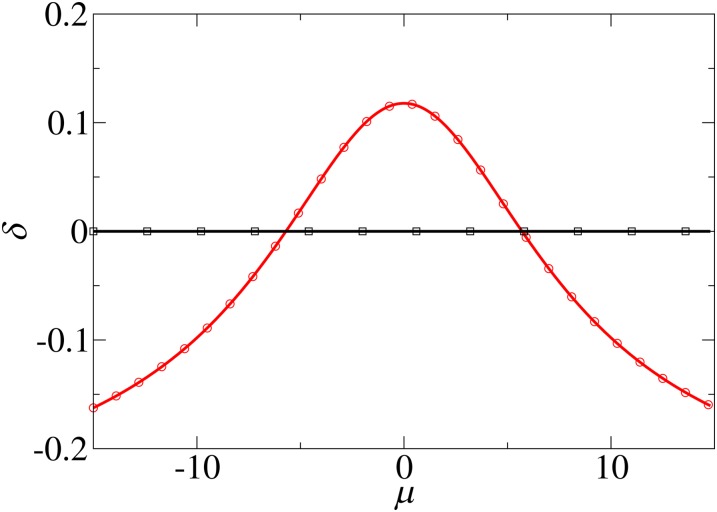
Gaussian selection function: deviation *δ* from theoretical prediction of the breeder’s and alternative equations as a function of the mean of the selection function *μ*. The breeding value distribution corresponds to equation (27). Black line (squares): (*R* − *μ*)/(*S* − *μ*) − *j*^2^; red line (circle): *R*/*S* − *h*^2^. Parameters used here are *σ_A_* = 2, *σ_W_* = 1 and *σ_E_* = 3 and therefore *h*^2^ = 4/13 and *j*^2^ = 10.

Figure 3 illustrates the accuracy of the alternative relation compared to the usual breeder’s equation for this case.

The aforementioned few examples were to illustrate the alternative breeder’s equation. Many other examples of the breeding value distributions, such as a double Gaussian or a rectangular function can be computed exactly and lead of course always to the alternative breeder’s equation. The last example can indeed be generalized and used as an alternative demonstration of equation (21), as any function can be seen as a superposition of Dirac’s deltas: $p_{0}\left(y\right)=\int_{\mathbb{R}}p_{0}\left(u\right)\delta \left(u-y\right)du$. The demonstration we provided using FT is, however, more straightforward.

### Selection on multiple traits

The results of the aforementioned sections are naturally generalized to selection on multiple traits. Consider the vectors of parental breeding values **y**_0_ = (*y*_1_, *y*_2_, …, *y_N_*), environmental effects **e** = (*e*_1_, …, *e_N_*) and their phenotype **z**_0_ = **y**_0_ + **e**, to which a selection function *W*(**z**) is applied. Using the same notations as in the previous sections, we find without difficulty that

$${\bar{z}}_{1}=\int_{{\mathbb{R}}^{N}\times {\mathbb{R}}^{N}}yp_{0}\left(y\right)W\left(z\right)f\left(z-y\right)dydz$$

$${\bar{z}}_{w}=\int_{{\mathbb{R}}^{N}\times {\mathbb{R}}^{N}}zp_{0}\left(y\right)W\left(z\right)f\left(z-y\right)dydz$$

As before, using FT, these relations transform into

$${\bar{z}}_{1}=\frac{i}{2\pi }\int_{{\mathbb{R}}^{N}}{\tilde{W}}^{*}\left(k\right)\left(\nabla {\tilde{p}}_{0}\left(k\right)\right)\tilde{f}\left(k\right)d^{M}k$$

$${\bar{z}}_{w}=\frac{i}{2\pi }\int_{{\mathbb{R}}^{N}}{\tilde{W}}^{*}\left(k\right)\nabla \left({\tilde{p}}_{0}\left(k\right)\tilde{f}\left(k\right)\right)d^{M}k$$

where ∇ is the gradient operator: ∇*f* = (∂*f*/∂*x*_1_, … ∂*f*/∂*x_N_*). We see again that ${\bar{z}}_{1}$ and ${\bar{z}}_{w}$ are linearly related if

$${\tilde{p}}_{0}\left(k\right)\left(\nabla \tilde{f}\left(k\right)\right)=A\left(\nabla {\tilde{p}}_{0}\left(k\right)\right)\tilde{f}\left(k\right)$$

where *A* is a constant matrix. The linear relation is automatically satisfied if both *p*_0_ and *f* follow a Gaussian distribution

$$p_{0}\left(y\right)\propto \text{exp}\left(-\frac{1}{2}y^{T}G^{-1}y\right)$$

$$f\left(x\right)\propto \text{exp}\left(-\frac{1}{2}x^{T}E^{-1}x\right)$$

where *G* and *E* are the covariance matrices for the genotype and environmental effects. Defining *P* = *G* + *E* as the phenotype covariance matrix, it is straightforward to show that in this case *A* = *EG*^−1^ and therefore (Lande 1979)

$${\bar{z}}_{1}=GP^{-1}{\bar{z}}_{w}$$

which is the usual breeder’s equation for multiple traits. We stress that the limitation of this relation is the same as that of the scalar version: it relies on the normal distribution of the genotype. On the other hand, if the selection function *W*(**z**) is Gaussian

$$W\left(z\right)\propto \text{exp}\left(-\frac{1}{2}{\left(z-\mu \right)}^{T}\Omega^{-1}\left(z-\mu \right)\right)$$

the arguments of the previous section 2 can be repeated and lead to the generalization of the alternative vectorial breeder’s equation (21)

$${\bar{z}}_{1}-\mu =\left(\Omega +E\right)\Omega^{-1}\left({\bar{z}}_{w}-\mu \right)$$

which, in analogy with equation (21) we write as

$$R^{\prime }=\left(\Omega +E\right)\Omega^{-1}S^{\prime }$$

## Discussion and Conclusion

The breeder’s equation is a cornerstone of quantitative genetics and appears as a fundamental equation in all the important textbooks of this field (Lynch and Walsh 1998; Falconer and Mackay 1995; Crow and Kimura 2009). It is widely used in artificial selection (Lush 1943; Hill and Kirkpatrick 2010); its usage in natural selection was popularized by Lande (1976), when he formalized the main idea of phenotypic evolution and it is now commonly used in many articles based on Lande’s work (see, for example, Hansen *et al.* 2011; Manna *et al.* 2011; Svardal *et al.* 2011). The mathematical foundation of this equation rests upon the hypothesis that the breeding value is normally distributed. This hypothesis is plausible for a continuous trait in a population not subject to selection (see, however, *Appendix/Segregation density function*). The normal distribution of the breeding value is more fragile in populations subjected to selection on this trait (Turelli and Barton 1990), as the genotype of selected parents is given by (equation 7)

$$p_{w}\left(y\right)=p_{0}\left(y\right).W^{†}\left(y\right)/\bar{W}$$

where *W*^†^(*y*) is the genotype fitness function defined by equation (8). Even if *p*_0_(*y*) were Gaussian, the very act of multiplying it by an arbitrary function makes *p_w_*(*y*), and hence *p*_1_(*y*) non-Gaussian. Therefore after the first round of selection, the normal distribution hypothesis of parental genotype cannot be sustained. Turelli and Barton (1994) have shown that for the infinitesimal model, the non-normality may not have large effects on the predictions of the breeder’s equation, but they argued that when the number of loci is limited the discrepancy can grow much larger. Of course even *p*_0_(*y*) cannot be assumed to be Gaussian if different breeds are crossed to constitute the parental generation, which happens in artificial selection and in natural selection when gene flow from nearby patches is important.

The breeding value is not an observable quantity. The fitness or selection function *W*(*z*) is more quantifiable and many authors have considered a Gaussian selection function. In artificial selection, it dates back at least to the work of Lush (Lush 1943), p140). In natural selection, it is used by most authors as a model for stabilizing selection. If Gaussian selection is used to evolve a population, then the alternative breeding equation (21) we derived is more precise and predictive and rests on more robust mathematical grounds while retaining the same simplicity of the standard breeder’s equation. Note that the analysis of this article is not restricted to the infinitesimal model, but applies to all inheritance processes involving purely additive genetic effects. The alternative breeder’s equation generalizes to selection on multiple traits in a way similar to the standard breeder’s equation and can therefore be incorporated in the “adaptive landscape” formalism (Arnold *et al.* 2001) with the same ease.

In conclusion, we believe that in all cases where Gaussian selection functions are used to evolve a population, the alternative breeder’s equation we develop above is a useful alternative approach to the standard method.

## Appendix

### FTs and convolutions

Logarithm was invented to simplify algebraic operations: a multiplication in the direct space transforms into an addition in the logarithm space, were it can be performed easily and the result brought back to direct space via the inverse transformation. FT plays a similar role in functional analysis, where derivation/integration of functions in direct space are transformed into multiplication/division by the variable in the Fourier space.

The FT of a function *f*(*x*) is defined here as (Byron and Fuller 1992)

$$\tilde{f}\left(k\right)=\text{TF}\left[f\left(x\right)\right]=\int_{-\infty }^{+\infty }f\left(x\right)e^{-ikx}dx$$

where *i*^2^ = −1. For example, the FT of the function *f*(*x*) = exp(−*α*|*x*|) is

$$\begin{array}{c} \tilde{f}\left(k\right)=\int_{-\infty }^{0}e^{\left(\alpha -ik\right)x}dx+\int_{0}^{\infty }e^{-\left(\alpha +ik\right)}dx\\ =\frac{1}{\alpha -ik}+\frac{1}{\alpha +ik}=\frac{2\alpha }{\alpha^{2}+k^{2}}. \end{array}$$

The main properties of FT we use here are

#### 1. Parseval’s theorem

$$\int_{-\infty }^{+\infty }f^{*}\left(x\right)g\left(x\right)dx=\frac{1}{2\pi }\int_{-\infty }^{+\infty }{\tilde{f}}^{*}\left(k\right)\tilde{g}\left(k\right)dk$$

where *a*∗ stands for the conjugate complex of *a*. Note that if *f*(*x*) is a real function, the ${\tilde{f}}^{*}\left(k\right)=\tilde{f}\left(-k\right)$, which ensures that the right hand side of the above expression is always real if both functions *f* and *g* are real.

#### 2. Derivation property

$$i\frac{d}{dk}\tilde{f}\left(k\right)=\int_{-\infty }^{+\infty }xf\left(x\right)e^{-ikx}dx=\text{TF}\left[xf\left(x\right)\right]$$

#### 3. Convolution property

$$\text{FT}\left[\left(f*g\right)\left(x\right)\right]=\text{FT}\left[f\left(x\right)\right].\text{FT}\left[g\left(x\right)\right]=\tilde{f}\left(k\right).\tilde{g}\left(k\right)$$

where

$$\left(f*g\right)\left(x\right)=\int_{-\infty }^{\infty }f\left(u\right)g\left(x-u\right)du$$

#### 4. Translation property

$$\text{FT}\left[f\left(x-\mu \right)\right]=e^{-ik\mu }\text{FT}\left[f\left(x\right)\right]$$

On the basis of the aforementioned properties, and the fact that all the aforementioned functions are real *i.e.*, for example *W*∗(*z*) = *W*(*z*), we see that relation (3) can be written as

$$\begin{array}{c} \iint_{{\mathbb{R}}^{2}}yp_{0}\left(y\right)W^{*}\left(z\right)f\left(z-y\right)dydz=\int_{\mathbb{R}}W^{*}\left(z\right)\left(yp_{0}*f\right)\left(z\right)dz\\ =\frac{i}{2\pi }\int_{\mathbb{R}}{\tilde{W}}^{*}\left(k\right)\frac{d}{dk}\left[\tilde{p}\left(k\right)\right]\tilde{f}\left(k\right)dk \end{array}$$

where we have used the fact (i) that $\mathrm{FT}\left[yp_{0}\left(y\right)\right]=i{\tilde{p}}^{\prime }\left(k\right)$ ; (ii) FT transforms a convolution product into a simple product in reciprocal space and (iii) Parseval’s theorem.

The same set of rules leads to

$$\begin{array}{c} \iint_{{\mathbb{R}}^{2}}zp_{0}\left(y\right)W^{*}\left(z\right)f\left(z-y\right)dydz=\int_{\mathbb{R}}W^{*}\left(z\right)z\left(p_{0}*f\right)\left(z\right)dz\\ =\frac{i}{2\pi }\int_{\mathbb{R}}{\tilde{W}}^{*}\left(k\right)\frac{d}{dk}\left[\tilde{p}\left(k\right)\tilde{f}\left(k\right)\right]dk \end{array}$$

Note that we can exchange the order of integration on *y* and *z*, write the first integral as a convolution product on functions of *z* and proceed to the second integral by using the FT on *y*. For *R*, we have

$$\begin{array}{c} \iint_{{\mathbb{R}}^{2}}yp_{0}\left(y\right)W\left(z\right)f\left(z-y\right)dydz=\int_{\mathbb{R}}p_{0}^{*}\left(y\right)y\left(W^{*}f\right)\left(y\right)dy\\ =\frac{i}{2\pi }\int_{\mathbb{R}}{\tilde{p}}^{*}\left(k\right)\frac{d}{dk}\left[\tilde{W}\left(k\right)\tilde{f}\left(k\right)\right]dk \end{array}$$

and for *S* we get

$$\begin{array}{c} \iint_{{\mathbb{R}}^{2}}zp_{0}\left(y\right)W\left(z\right)f\left(z-y\right)dydz=\int_{\mathbb{R}}p_{0}^{*}\left(y\right)\left(zW*f\right)\left(y\right)dy\\ =\frac{i}{2\pi }\int_{\mathbb{R}}{\tilde{p}}^{*}\left(k\right)\frac{d}{dk}\left[\tilde{W}\left(k\right)\right]\tilde{f}\left(k\right)dk \end{array}$$

The translation property was used in the derivation of the functional lags (eqs 19,20).

Finally, note that the FT of a Gaussian is a Gaussian:

$$\mathrm{FT}\left[\frac{1}{\sqrt{2\pi }s}\exp(-x^{2}/\left(2s^{2}\right)\right]=\exp\left(-s^{2}k^{2}/2\right)$$

### Computation of truncation selection with FT

Consider a normal breeding value and environmental factor distribution

$$p_{0}\left(y\right)=N\left(0,\sigma_{A};y\right)$$

$$f\left(x\right)=N\left(0,\sigma_{E};x\right)$$

where $N\left(a,b;u\right)=\left(1/\sqrt{2\pi }b\right)\exp\left(-{\left(u-a\right)}^{2}/2b^{2}\right)$ to which we apply a truncation selection *W*(*z*), where *W*(*z*) = 1 if *z*_0_ < *z* < *z*_1_ and 0 other wise. The FT of these functions read:

$${\tilde{p}}_{0}\left(k\right)=e^{-\sigma_{A}^{2}k^{2}/2}$$

$$\tilde{f}\left(k\right)=e^{-\sigma_{E}^{2}k^{2}/2}$$

$$\tilde{W}\left(k\right)=\frac{i}{q}\left(e^{-iqz_{1}}-e^{-iqz_{0}}\right)$$

The mean fitness of the parental generation is

$$\begin{array}{c} \bar{W}\left(z_{1},z_{0}\right)=\iint_{{\mathbb{R}}^{2}}p_{0}\left(y\right)W\left(z\right)f\left(z|y\right)dydz\\ =\frac{1}{2\pi }\int_{\mathbb{R}}{\tilde{W}}^{*}\left(k\right){\tilde{p}}_{0}\left(k\right)\tilde{f}\left(k\right)dk\\ =\frac{1}{2}\left(\mathrm{erf}\left(\frac{z_{1}}{\sqrt{2}\Delta }\right)-\mathrm{erf}\left(\frac{z_{0}}{\sqrt{2}\Delta }\right)\right) \end{array}$$

where $\Delta^{2}=\sigma_{A}^{2}+\sigma_{E}^{2}$ and erf(*u*) is the error function. As

$$\frac{d}{dk}\left[e^{-s^{2}q^{2}/2}\right]=-s^{2}qe^{-s^{2}q^{2}/2}$$

the computation of *R* and *S*, using their expression (13,14) is simple (the integrands are exact differentials) and leads to

$$R=\frac{1}{\bar{W}}\frac{\sigma_{A}^{2}}{\sqrt{2\pi }\Delta }\left(e^{-z_{0}^{2}/2\Delta^{2}}-e^{-z_{1}^{2}/2\Delta^{2}}\right)$$

$$S=\frac{1}{\bar{W}}\frac{\Delta }{\sqrt{2\pi }}\left(e^{-z_{0}^{2}/2\Delta^{2}}-e^{-z_{1}^{2}/2\Delta^{2}}\right)$$

and it is trivially verified that $R/S=\sigma_{A}^{2}/\Delta^{2}=h^{2}$.

The same computations can be extended to the case where the breeding value distribution is a double Gaussian (Figure 2):

$$p_{0}\left(y\right)=\frac{1}{2}\left(N\left(-m,s;y\right)+N\left(m,s;y\right)\right)$$

$${\tilde{p}}_{0}\left(k\right)=\frac{1}{2}\left(e^{imk}+e^{-imk}\right)e^{-s^{2}k^{2}/2}$$

The computations, although more cumbersome, involve the same level of technicality. For simplicity, we give the result only for extreme truncation *z*_1_ →∞ (selecting all individuals with phenotype > *z*_0_). Noting

$$\bar{W}\left(m\right)=\frac{1}{2}\left(1-\mathrm{erf}\left(\frac{z_{0}-m}{\sqrt{2}\Delta }\right)\right)$$

$$A\left(m\right)=\frac{s^{2}}{\sqrt{2\pi }\Delta }e^{-{\left(z_{0}-m\right)}^{2}/2\Delta^{2}}+\frac{m}{2}\mathrm{erfc}\left(\frac{z_{0}-m}{\sqrt{2}\Delta }\right)$$

$$B\left(m\right)=\frac{\Delta }{\sqrt{2\pi }}e^{-{\left(z_{0}-m\right)}^{2}/2\Delta^{2}}+\frac{m}{2}\mathrm{erfc}\left(\frac{z_{0}-m}{\sqrt{2}\Delta }\right)$$

Where $\Delta =s^{2}+\sigma_{E}^{2}$. The selection and response function read:

$$R=\frac{A\left(m\right)+A\left(-m\right)}{\bar{W}\left(m\right)+\bar{W}\left(-m\right)}$$

$$S=\frac{B\left(m\right)+B\left(-m\right)}{\bar{W}\left(m\right)+\bar{W}\left(-m\right)}$$

The simple Gaussian case can be recovered from these expressions by *m* = 0 and *σ_A_* = *S*.

### Parent-offspring regression

The derivation of the breeder’s equation sometimes uses the parent-offspring regression coefficient as an intermediate(Nagylaki 1992; Lynch and Walsh 1998). The linear regression between parent and offspring phenotype however is based on the same assumption of normal distribution of genotype and environmental factors.

The probability density of observing the phenotype *z*′ in the offspring and *z_a_*, *z_b_* in the parents is

$$\begin{array}{c} p\left(z^{\prime };z_{a},z_{b}\right)=\iint_{{\mathbb{R}}^{2}}p\left(y_{a}\right)f\left(z_{a}|y_{a}\right)p\left(y_{b}\right)f\left(z_{b}|y_{b}\right)L\left(y_{1}-\left(y_{a}+y_{b}\right)/2\right)f\left(z^{\prime }|y_{1}\right)dy_{1}dy_{a}dy_{b} \end{array}$$

and the conditional expectation of *z*′ given *z* is

$$E\left(z^{\prime }|z_{a},z_{b}\right)=\int_{z^{\prime }\in I}z^{\prime }p\left(z^{\prime },z\right)dz^{\prime }/\int_{z^{\prime }\in I}p\left(z^{\prime },z\right)dz^{\prime }=F\left(z_{a},z_{b}\right)$$

It is not difficult to check that the function *F*(*z_a_*, *z_b_*) is a linear function of its argument

$$F\left(z_{a},z_{b}\right)=b\left(z_{a}+z_{b}\right)/2$$

if both the genotype and environment factors obey a normal distribution, in which case, the linearity coefficient is indeed $b=\sigma_{A}^{2}/\left(\sigma_{A}^{2}+\sigma_{E}^{2}\right)$. However, *even* if the parental generation follows a normal distribution, the *selected* parents do not (equation 7) and the use of parent-offspring regression poses even more of a problem than the direct method.

### Segregation density function

Let *p*_0_(*y*) be the distribution of breeding value in the parental generation. In the absence of selection, after recombination-segregation, the distribution of breeding value in the progeny is

$$p_{1}\left(y\right)=\iint_{{\mathbb{R}}^{2}}p_{0}\left(y_{a}\right)p_{0}\left(y_{b}\right)L\left(y-\left(y_{a}+y_{b}\right)/2\right)dy_{a}dy_{b}$$ (28)

where the function *L*(*y*) is the segregation density function capturing the inheritance process of the breeding value (Karlin 1979). *L*(*y*) is a probability density function and in the absence of epistasis and dominance effect, its average is zero: $\int_{\mathbb{R}}yL\left(y\right)dy=0$. In the infinitesimal model framework, *L*(*y*) is a normal distribution of variance $\sigma_{A}^{2}/2$ . However, any distribution probability *L*(*y*) will lead to a stable, although not necessarily normal, probability distribution of breeding values after few round of reproduction. Let us set the origin of the breeding value at its average in the parental distribution, *i.e.* $\int_{\mathbb{R}}yp_{0}\left(y\right)dy=0$. In Fourier space relation (28) is

$${\tilde{p}}_{1}\left(k\right)={\tilde{p}}_{0}^{2}\left(k/2\right)\tilde{L}\left(k\right)$$

and after *n* rounds of reproduction,

$${\tilde{p}}_{n}\left(k\right)={\tilde{p}}_{0}^{2^{n}}\left(k/2^{n}\right)\prod_{i=0}^{n-1}{\tilde{L}}^{2^{i}}\left(k/2^{i}\right)$$

As both *p*_0_(*y*) and *L*(*y*) are probability distribution functions of zero mean, we have

$${\tilde{p}}_{0}\left(0\right)=\tilde{L}\left(0\right)=1$$

$${\tilde{p}}_{0}^{\prime }\left(0\right)={\tilde{L}}^{\prime }\left(0\right)=0$$

and therefore

$${\tilde{p}}_{n}^{\prime \prime }\left(0\right)=\frac{1}{2^{n}}{\tilde{p}}_{0}^{\prime \prime }\left(0\right)+\left(2-\frac{1}{2^{n-1}}\right){\tilde{L}}^{\prime \prime }\left(0\right)$$

Let $V=\int_{\mathbb{R}}y^{2}L\left(y\right)dy$ . We see then that

$$\mathrm{Var}\left(Y_{n}\right)=\frac{1}{2^{n}}\mathrm{Var}\left(Y_{0}\right)+\left(2-\frac{1}{2^{n-1}}\right)V$$

So the variance of the breeding values converges fast to twice the variance of the segregation density function. The distribution function *p_n_*(*y*), however, converges to a normal distribution only if *L*(*y*) is normal.
